# Artemisone effective against murine cerebral malaria

**DOI:** 10.1186/1475-2875-9-227

**Published:** 2010-08-09

**Authors:** Judith H Waknine-Grinberg, Nicholas Hunt, Annael Bentura-Marciano, James A McQuillan, Ho-Wai Chan, Wing-Chi Chan, Yechezkel Barenholz, Richard K Haynes, Jacob Golenser

**Affiliations:** 1 Department of Microbiology and Molecular Genetics, The Hebrew University of Jerusalem, Jerusalem, 91120, Israel; 2 Laboratory of Membrane and Liposome Research, Department of Biochemistry, Institute for Medical Research - Israel-Canada, The Hebrew University of Jerusalem - Hadassah Medical School, Jerusalem, Israel; 3 Department of Pathology and Bosch Institute, The University of Sydney, Sydney, Australia; 4 Department of Chemistry, Institute of Molecular Technology for Drug Discovery and Synthesis, The Hong Kong University of Science and Technology, Clear Water Bay, Kowloon, Hong Kong

## Abstract

**Background:**

Artemisinins are the newest class of drug approved for malaria treatment. Due to their unique mechanism of action, rapid effect on Plasmodium, and high efficacy in vivo, artemisinins have become essential components of malaria treatment. Administration of artemisinin derivatives in combination with other anti-plasmodials has become the first-line treatment for uncomplicated falciparum malaria. However, their efficiency in cases of cerebral malaria (CM) remains to be determined.

**Methods:**

The efficacy of several artemisinin derivatives for treatment of experimental CM was evaluated in ICR or C57BL/6 mice infected by *Plasmodium berghei *ANKA. Both mouse strains serve as murine models for CM.

**Results:**

Artemisone was the most efficient drug tested, and could prevent death even when administered at relatively late stages of cerebral pathogenesis. No parasite resistance to artemisone was detected in recrudescence. Co-administration of artemisone together with chloroquine was more effective than monotherapy with either drug, and led to complete cure. Artemiside was even more effective than artemisone, but this substance has yet to be submitted to preclinical toxicological evaluation.

**Conclusions:**

Altogether, the results support the use of artemisone for combined therapy of CM.

## Background

**Figure 1:**
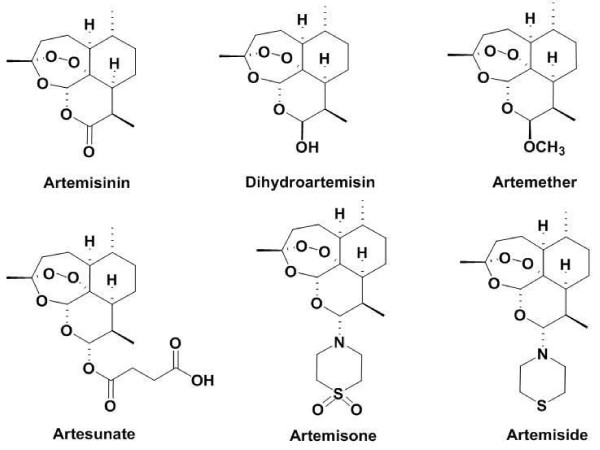
**Structures of artemisinin and its derivatives**.

The most critical problem currently limiting malaria treatment is the emergence and spread of parasite resistance to the majority of anti-malarial drugs in use [1]. Improper or incomplete monotherapy of malaria has caused the development of resistance to the commonly used chloroquine [2,3] and mefloquine [4], and even to quinine, which has been a mainstay in the anti-malarial pharmacopeia for approximately two centuries [5]. Artemisinin derivatives comprise the most recently developed class of anti-malarial drugs currently approved for human use. These derivatives (Figure 1) include artesunate and artemether, their metabolite dihydroartemisinin (DHA), and artemisone [6,7]. All artemisinins comprise a peroxide bridge, essential for activity, embedded within the 1,2,4-trioxane unit in a fused tetracyclic sesquiterpene scaffold.

Artemisinin-type drugs have been proposed to act via several mechanisms. A widely held view is that ferrous iron, either 'free' or in haemoglobin (Hb), or its breakdown product, haem, is required for activation of the peroxide [8-11]. Fenton chemistry involving reductive cleavage of the peroxide by Fe(II) is considered to lead to C-centered radicals that are the presumed cytotoxic agents [12]. For haem, it is assumed that the C-radicals alkylate the haem nucleus to provide adducts that discharge the potent activities of the parent artemisinin [13,14]. However, artemisinins susceptible to decomposition by haem-Fe(II) display enhanced activities against parasites cultured under carbon monoxide (CO), an agent that passivates haem-Fe(II) by formation of stable haem-Fe(II)-CO complexes; this thereby discounts haeme as an activator of artemisinins [15,16]. Artemisinins are known mediators of oxidative stress [17] that enhance oxidative stress in malaria parasites [18,19]. Oxidative damage has been observed to occur in various parasite membranes [6], the mitochondria [20] and DNA [21]. Another view, based on the observation that in vitro anti-malarial activity is sensitive to steric effects, suggests that the molecule undergoes activation after binding to a specific protein target site. Artemisinins have been shown to interfere with the activity of the *Plasmodium falciparum *sarcoplasmic/endoplasmic calcium ATPase (SERCA), PfATP6 [22]. SERCA is responsible for the maintenance of calcium ion concentrations, which is important for the generation of calcium-mediated signalling as well as for the correct folding and post-translational processing of proteins. Artemisinins also inhibit endocytosis by the parasite. Although no direct link has been reported, changes in cytosolic Ca^+2 ^levels as a result of SERCA inhibition may have a significant regulatory effect on endocytosis [23].

The question of plasmodial resistance to artemisinins has been a matter of debate [6]. However, there is growing evidence that uncontrolled (non-regulated) use of these drugs is followed by reduced susceptibility and resistance [24-26]. An increase in parasite cytochrome P450 and MDR1 activities may also be the cause of reduced drug efficacy [27]. Artemisinin-based combination therapy (ACT) is recommended for more efficient treatment and for prevention of the induction of drug resistance [28-32]. A recent review [31] describes improved results when ACT is used, compared to non-artemisinin-based combinations. Most of the studies described were performed using artesunate and were directed against uncomplicated falciparum malaria.

The search for new anti-malarial therapeutics has been directed towards identification of new drug targets in the parasite. As many aspects of the disease and its complications are caused by the host anti-malarial immune response, this approach is incomplete. Cerebral malaria (CM), one of the most serious complications of *P. falciparum *infection, affects mainly children and non-immune adults [33]. In areas of high transmission young children are more affected by severe malaria, including CM, as immunity is achieved only after a prolonged period of exposure, while in low transmission areas CM is expressed in older children. The pathogenesis of CM remains unclear. One explanation is adherence and sequestration of parasitized erythrocytes, peripheral blood mononuclear cells (PBMC) and platelets to vascular endothelial cells lining the small blood vessels of the brain [34]. However, there is no direct proof in humans for a causative relationship between sequestrations of PBMC and the pathogenesis of CM. Studies using a murine model of experimental cerebral malaria (ECM) with *Plasmodium berghei *ANKA indicate that ECM is a T cell-mediated disease. In particular, CD8^+ ^cytotoxic T cells are involved in the destruction of the blood-brain barrier (BBB) by perforin-dependent processes [35-38]. Overall, parasite-triggered cerebral inflammation is considered to be a possible cause of death from CM [39,40]. The harmful, dysregulated immune response leading to CM or ECM is mainly of the Th1 type, with overproduction of some cytokines, such as IFNγ (initially produced by Th1 cells, and at a later stage also by CD8^+ ^cytotoxic T cells), combined with underproduction of others (e.g. IL-10) [41]. Both sequestration and inflammation are needed for induction of cerebral pathogenesis [40]. A preventive measure that will eliminate or reduce even a single factor associated with the cascade of events leading to (E)CM may completely avert it [6,42]. In addition to their anti-plasmodial activity, artemisinins have been shown to reduce CD4^+ ^and CD8^+ ^T cell inflammatory responses [43-46]. The severity of malarial infection is correlated with both brain endothelial cell activation and blood brain barrier disruption [47,48]. Vascular endothelial growth factor (VEGF) activation of vascular and endothelial cells causes disruption of BBB function as well as angiogenesis in blood vessels where parasite sequestration has occurred. This results in haemorrhages and fatal brain oedema, and is thought to be the source of the neurological impairments seen in those that survive the infection [49,50]. Artemisinins have been shown to down-regulate VEGF functions [51,52]; VEGF down-regulation may alleviate the symptoms of CM and reduce the neurological damage in those who survive the disease [6].

It is apparent that artemisinins, including artemisone, will be useful for the treatment of CM. Artemisone is non-neurotoxic, possesses anti-malarial activities greater than those of artesunate, remains active in the presence of Hb-Fe(II), does not alkylate haem-Fe(II) under biologically plausible conditions, and is unaffected by free Fe(II). In CM, where abnormal quantities of Hb-Fe(II), haem-Fe(II), or free Fe(II) are present [53], artemisone will be less prone to competitive degradation.

Thus, it is clear that the suitability of artemisinin-type drugs for CM prophylaxis and/or therapy merits close examination. Our research demonstrates the effects of several artemisinin derivatives on the progression of murine malaria, with an emphasis on artemisone therapy for ECM. The possible induction of artemisone resistance and the efficiency of artemisone monotherapy vs. combined artemisone-chloroquine treatment is also examined.

## Methods

### Mice

ICR Harlan-Sprague-Dawley (ICR) or C57Bl/6 Ola-Hsd male mice (C57Bl/6, from Harlan, Israel; C57Bl/6Aus from the Animal Resource Centre, Australia:) aged 7-8 weeks were used in all experiments, 6 to 10 mice per group (as described). The mice were housed under standard light and temperature conditions and provided with unlimited access to water and food. All experiments were carried out in accordance with institutional guidelines for animal care, by protocols approved by the Animal Ethical Care Committee of The Hebrew University of Jerusalem or the University of Sydney. Parasitaemia was monitored every other day, from the time of inoculation until death or sacrifice, by thin blood smears prepared from tail blood. These were stained with a 25% Giemsa solution, examined under a light microscope, and parasitaemia determined as the number of infected red blood cells per 10,000 erythrocytes. Clinical signs of cerebral malaria were evaluated and used for scoring disease severity (Table 1).

**Table 1:** Scoring chart for definition and severity of ECM disease as per clinical signs.

Appearance	Normal	0
	Coat ruffled	1
	Coat staring; panting	2
Behaviour (undisturbed)	Normal	0
	Hunched; wobbly gait	1
	Partial paralysis; immobile*^†^	2
	Convulsions; coma*	3

Food intake	Normal	0
	Up to 10% loss in body weight	1
	10%-15%* loss in body weight	2
	More than 15% loss in body weight*	3

Body temperature	Normal (36-37°C)	0
	34-35°C	1
	32-33°C*	2
	Below 32°C*	3

Mice with a cumulative score of 5 or above are sacrificed, and death deemed to be on the following day.

*Endpoint for mouse sacrifice.

^†^Also in response to stimulation.

### Parasites

*Plasmodium berghei *ANKA (PbA MRA-311, CDC Atlanta) was maintained in vivo by serial transfer of parasitized erythrocytes (PE) from infected to naïve mice. Experimental mice were infected by intraperitoneal (i.p.) injection of 5 × 10^4 ^PE from peripheral blood of infected donor mice, an inoculum which caused fatal ECM in at least 50% of infected ICR HSD mice, and 90% of infected C57Bl/6 Ola-Hsd mice. A significant increase in the inoculum level reduced the incidence of ECM [54].

In experiments performed at the University of Sydney, *P. berghei *ANKA (designated PbAus in the text; courtesy of Prof. G. Grau, University of Sydney, Australia), was injected to C57Bl/6Aus mice at an inoculum of 10^6 ^PE. Inoculation of C57Bl/6 mice with PbAus is a well described model, in which the rate of fatal ECM is at least 90% [55].

The link between death and ECM in mouse models has previously been demonstrated [41,56]. Observed clinical symptoms, including an accelerated drop in core body temperature and death at low parasitaemias, indicate ECM [48,57,58]. Scoring of disease severity was performed in order to track the development of ECM, according to the severity levels described in Table 1. Infected mice were evaluated for parasitaemia levels, the appearance of neurological symptoms and changes in weight and temperature. Clinical signs of neuropathology that appear 1-2 days before death from ECM include marked coat staring, hunching, wobbly gait and reduced locomotion, convulsions, and coma. Mice that died at a parasitaemia of 15% or below with accompanying neurological symptoms and drastic reductions in body weight and temperature were considered to have died of ECM. ECM death generally occurred on days 8-10 post-inoculation. Brain pathology observed in mice dying of ECM includes haemorrhages, mononuculear cell accumulation in small vessels and the development of brain oedema. Untreated mice which did not die from ECM went on to die with severe anaemia and hyperparasitaemia, as has been reported in all other cases where mice are resistant to *P. berghei *ANKA-induced ECM [59].

### Drugs

DHA and artesunate were purchased from the Kunming Pharmaceutical Corporation via the Hong Kong University of Science and Technology, and used as received. Artemisone and artemiside were synthesized from DHA and purified by flash column chromatography, followed by recrystallization according to the procedure previously reported [7]. Dimethyl sulfoxide (DMSO) and chloroquine diphosphate were purchased from Sigma-Aldrich, Ltd (Israel). All artemisinin derivatives were prepared in DMSO according to the required dosage and administered in a volume of 20-50 μl by intraperitoneal injection. Chloroquine diphosphate was dissolved in PBS and administered in a 50 μl volume by intraperitoneal injection.

### Histology

Mice were deeply anaesthetized and sacrificed by terminal intracardial perfusion with 10 ml ice-cold PBS. Organs were removed and fixed overnight in 10% (v/v) neutral buffered formalin (Fronine, Australia). Paraffin-embedded tissues were cut into 5-7 μm slices, deparaffinated, and stained with haematoxylin and eosin before coverslipping.

### Statistics

When comparing parasitaemia values, p values were calculated using Students t-test; for analysis of survival curves, the Kaplan-Meier test was employed. In both cases, values below 0.05 were considered significant.

## Results

The general approach was to establish the lowest dose required to save the mice from ECM, using the artemisinin derivatives. Per the results, the most effective drug was chosen and used to determine the latest day post-inoculation (as close as possible to the time of overt ECM expression) at which treatment was still efficient. Following results depicting recrudescence, experiments were performed to determine whether recrudescent parasites displayed any resistance to the drugs used for treatment. Finally, combination therapy was administered to prevent recrudescence and achieve complete cure.

Mice were treated with the artemisinin derivatives dissolved in DMSO or administered the same volume of DMSO as a control, on different days post-inoculation. Injection of DMSO itself may partially reduce ECM and shift the pattern of disease to anemic malaria (see below). Table 2 depicts the results of an experiment in which PbA-infected female ICR mice were treated by i.p. injection of artemisinin derivatives, once a day on days 2-5 post-inoculation. No significant differences in the pattern of disease development were seen as a result of treatment, except in the case of artemiside; administration of this drug resulted in complete cure. These results also suggest that treatment with artemisinins would necessitate more than one injection per day. Despite the superior effect of artemiside, the experiments were continued using artemisone, as it has been already approved for human use [60].

**Table 2:** The effect of artemisinin derivatives on the development of experimental cerebral malaria in PbA-infected ICR mice.

Compound	Dose	% ECM	Delay to ECM (relative to control)
DMSO	50 μl	50-90%*	-
DHA	5 mg/kg/d	90%	-
	10 mg/kg/d	70%	-
	20 mg/kg/d	60%	2 days
Artesunate	2 × 5 mg/kg/d	65%	9 days**
Artemisone	5 mg/kg/d	100%	2 days
Artemiside	3 mg/kg/d	Total cure, no ECM**^,†^	-

Mice (n = 10 mice/group) were treated for two to five days post-inoculation. Death from ECM was apparent in control mice starting on day 7 post-inoculation. Mice which did not die of ECM died of severe anemic malaria.

* In 3 experiments.

** Significant

^† ^No recrudescence

**Figure 2:**
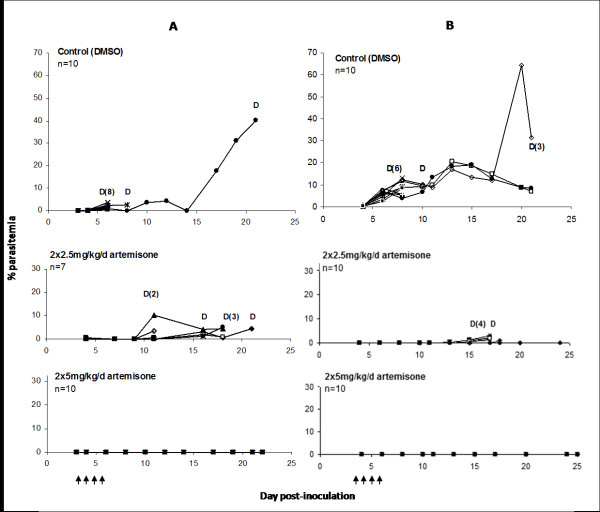
**Development of parasitemia in ICR (A) or C57Bl/6 (B) mice infected with PbA and treated with 2 × 2.5 or 2 × 5 mg/kg/d artemisone on days 3-6 post-inoculation**. D notes mouse death; multiple deaths on a given day are noted in parentheses. Each line represents a single mouse; arrows denote treatment. ECM death in both control groups occurred on days 8-10 post-inoculation. Administration of 2 × 2.5 mg/kg/d artemisone was not curative in ICR mice, but led to complete cure in 50% of C57Bl/6 mice; treatment with 2 × 5 mg/kg/day led to complete cure in both mouse strains.

**Figure 3:**
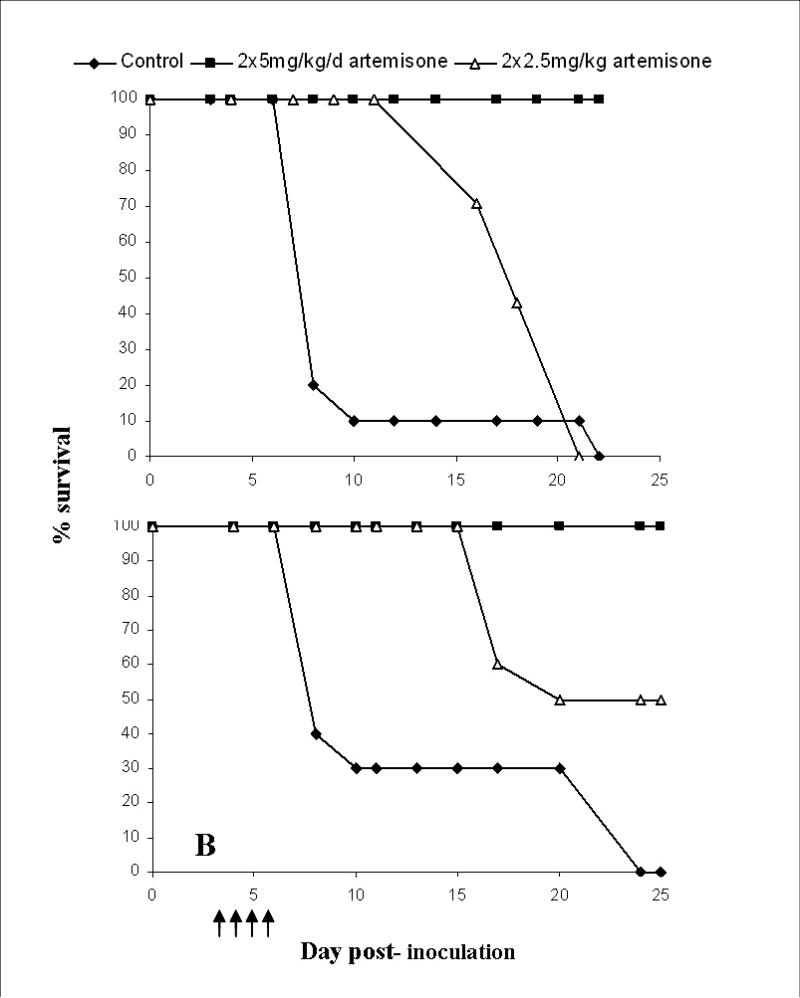
**Survival of ICR (A) or C57Bl/6 (B) mice infected with PbA and administered 2 × 2.5 or 2 × 5 mg/kg/d artemisone on days 3-6 post-inoculation**. Significant differences in survival were seen between control and treated groups, in both ICR and C57Bl/6 mice (p < 0.01): artemisone treatment delayed mouse death by up to two weeks. Arrows denote treatment.

In the next experiment (Figure 2), artemisone was administered i.p. as a split daily dose on days 3-6 post-inoculation, to either ICR or C57BL/6 mice infected with PbA. 90% of control ICR mice died of ECM by day 8; all mice treated with 2 × 2.5 mg/kg/d artemisone relapsed after an initial drop in parasitemia and died of ECM, while the higher dose (2 × 5 mg/kg/d artemisone) was curative in all treated mice (Figure 2A). In C57Bl/6 mice, 70% of DMSO-treated control mice died of ECM. Administration of 2 × 2.5 mg/kg/d artemisone led to rapid parasite clearance and complete cure in 50% of treated mice; recrudescence was observed on day 13 post-inoculation in the remaining mice, which died of ECM (Figure 2B). Treatment with 2 × 5 mg/kg/day led to complete cure in both ICR and C57Bl/6 mice. The differences in survival between all three groups (control vs. treated, and 2 × 2.5 vs. 2 × 5 mg/kg/d C57Bl/6 mice) were significant (p < 0.01). Artemisone treatment significantly reduced parasitemias (p < 0.001) and increased survival times, delaying mouse death by up to two weeks (Figure 3). In ICR mice, the increased survival time of mice treated with 2 × 5 mg/kg/d artemisone, compared to control mice, was statistically significant (p < 0.001), as was the difference in survival time when treated groups were compared (p < 0.001).

**Figure 4:**
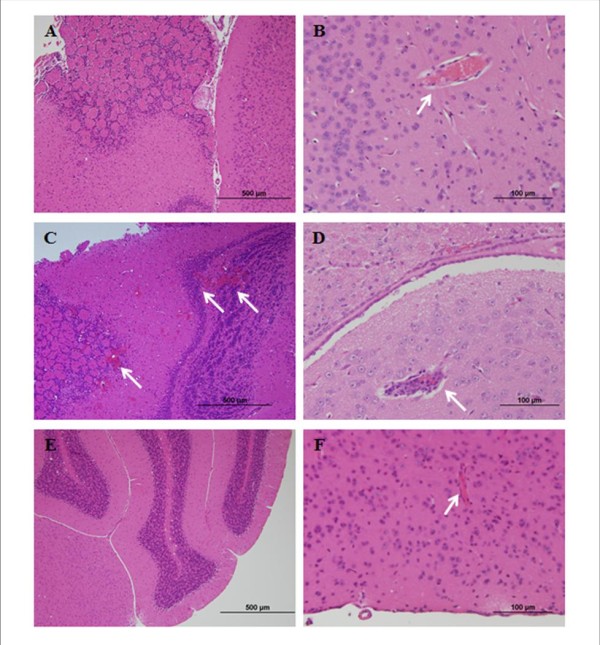
**Brain histopathology of non-infected C57Bl/6Aus mice (A, B), PbAus-infected C57Bl/6Aus mice on day 6 post-inoculation (C, D), or infected artemisone-treated C57Bl/6Aus mice on day 8 (E) or 13 (F) post-inoculation**. All PbAus-infected control mice died of ECM on days 7-12 post-inoculation. PbAus-infected mice treated with 2 × 20 mg/kg/d artemisone on days 6-8 post-inoculation were sacrificed on days 8-13 post-inoculation and brains taken for histology. Histological analysis of brain sections from non-infected mice demonstrated healthy parenchyma and intact blood vessels. Haemorrhages and leukocyte-packed vessels were apparent in mice one week post-PbAus inoculation while artemisone-treated mice displayed only minor haemorrhaging; vessels were intact and clear of leukocytes. Arrows indicate hemorrhages and/or blood vessels.

Table 3 presents a summary of experiments performed to examine the effect of treatment timing on the course of the disease. C57Bl/6Aus mice were infected with PbAus and administered artemisone according to different schedules. Control mice were divided into two groups: one group was not treated, while the other was injected with DMSO in parallel to the drug treatment schedule. 5/6 non-injected control mice died of ECM on days 6-9 post-inoculation; DMSO treatment for six successive days (2 × 25 μl/day on days 3-7 and 1 × 25 μl/day on day 8 post-inoculation) reduced the occurrence of ECM to 3/6 mice (Table 3, groups 1 and 2). Mice that did not succumb to ECM died of severe anemic malaria, three weeks later and at high parasitaemias (above 50%). For the drug-treated groups, the start of treatment was determined according to parasitaemia levels: injections began on day 3 or 4 post-inoculation, when the parasitaemia reached 1%. Treatment with 2 × 5 mg/kg/day artemisone on days 3-7 or 4-7, followed by a single injection (5 mg/kg/day) on day 8 post-inoculation, reduced parasitaemias to below detection level and led to complete cure in the majority of the mice. In each group, one mouse recrudesced after 10 or 11 days, developed high parasitaemia and died of severe anaemic malaria. If treatment was further delayed to days 5-8 post-inoculation, there was a typical latent period followed by complete cure (2/5 mice) or recrudescence and death from severe anaemic malaria (3/5 mice) (Table 3, group 5). No ECM deaths due to recrudescence were observed. Furthermore, injection of infected mice with 2 × 20 mg/kg/d artemisone at a very late stage of the disease, on days 6-8 post-inoculation (when parasitaemia was ~5%), completely eliminated the parasites and prevented ECM in all treated mice. The choice of dose was based on experiments in which 2 × 5 mg/kg was curative (see below): 2 × 20 mg/kg was used as a measure for the margin of safety and for proof of concept - that a non-toxic dose can prevent neuropathology. 6/7 control untreated mice died of ECM on day 7; the remaining mouse died of ECM on day 8. Histological analysis of brain sections from non-infected mice (Figure 4A) demonstrated healthy parenchyma and intact blood vessels. Neuropathological changes, namely haemorrhages and leukocyte-packed vessels, were apparent in mice one week post-PbAus inoculation (Figure 4B). In contrast, artemisone-treated mice (Figure 4C) displayed only minor haemorrhaging; vessels were intact and clear of leukocytes.

**Table 3:** Disease pattern in PbAus-infected C57BL/6Aus mice treated with 2 × 5 mg/kg/d rtemisone.

Group	Compound	Treatment* (days post-inoculation)	Disease pattern			
			Latency, days	Cerebral malaria	Anemic malaria	Cured**
1	-	-	0	5/6	1/6	-
2	DMSO (25 μl)	3-8	0	3/6	3/6	-
3	Artemisone	3-8	10 (n = 1)^†^	0/6^†^	1/6	5/6^†^
4	Artemisone	4-8	11 (n = 1) ^†^	0/6^†^	1/6	5/6^†^
5	Artemisone	5-8	10 (n = 4) ^†^	0/5^†^	3/5^‡^	2/5^†,‡^

*2 × 5 mg/kg/d every day except day 8 (1 injection, 5 mg/kg/d).

** No recrudescence

^†^Significant vs. control

^‡^Significant vs. other artemisone-treated groups

**Figure 5:**
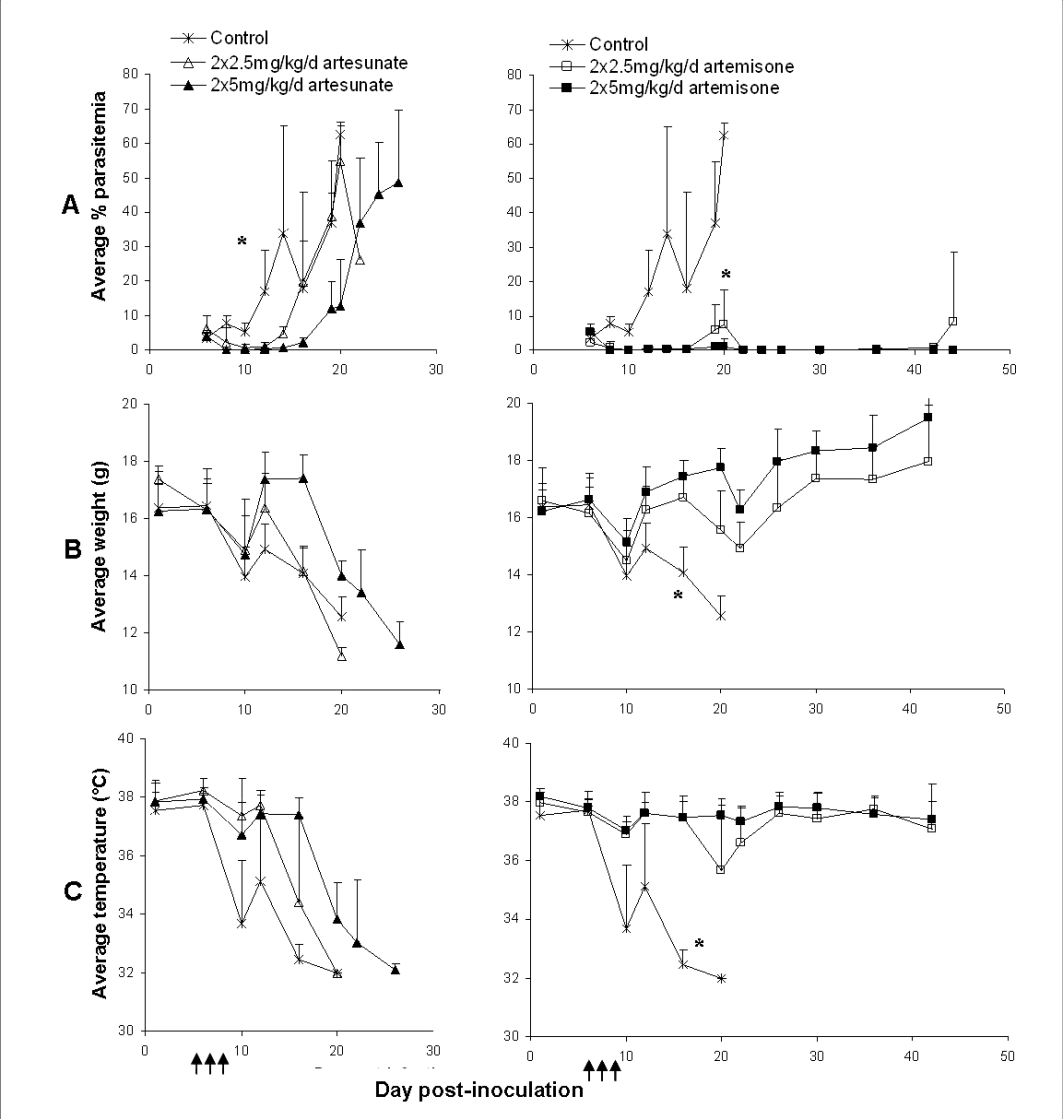
**Parasitemias (A), weights (B), and body temperatures (C) of PbAus-infected C57Bl/6 mice treated with 2 × 2.5 mg/kg/d or 2 × 5 mg/kg/d artesunate or artemisone**. Injections (arrows) were given on days 7-9 post-inoculation; control mice were administered 2 × 20 μl DMSO per day. *p < 0.001, control vs. treated mice. Administration of 2 × 2.5 mg/kg/d artesunate did not prevent ECM death; 2 × 5 mg/kg/d artesunate prevented ECM in most mice and delayed death from anemic malaria by approximately one week. Both doses of artemisone prevented ECM in all treated mice, delayed recrudescence and prevented death. Changes in temperature and weight were in accordance with disease severity, and the differences in overall group scores. Average values ± SD (error bars) are presented.

**Figure 6:**
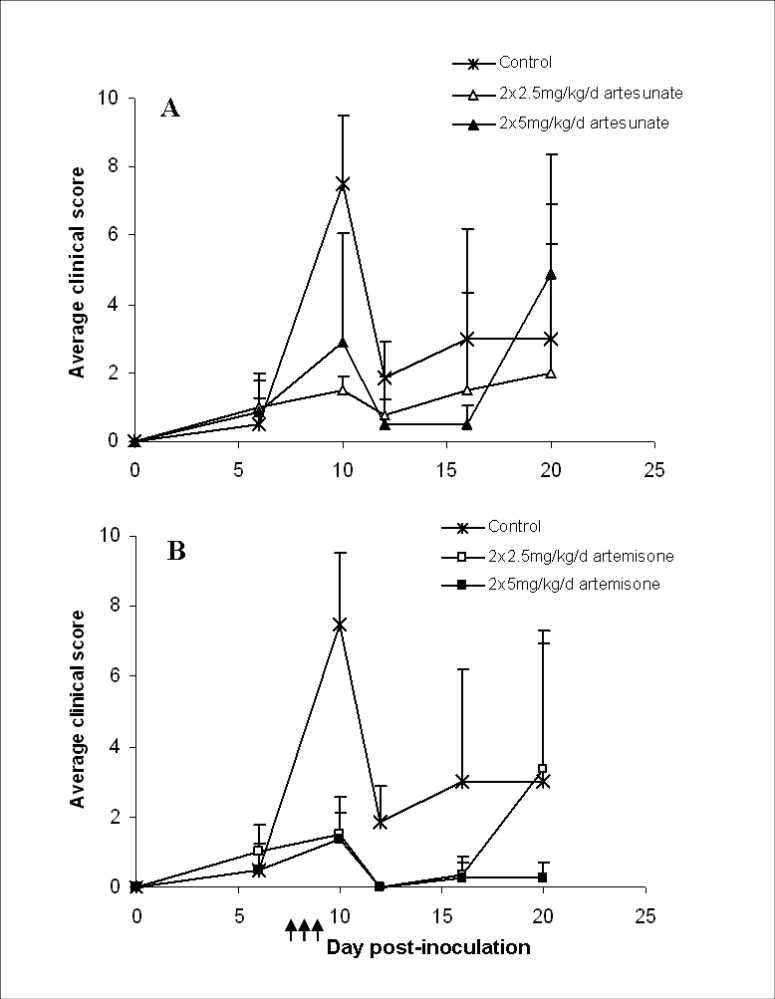
**Overall group scores for PbAus-infected C57BL/6 mice treated with artesunate (A) or artemisone (B), twice a day on days 7-9 post-inoculation**. Significant differences were seen between the control group and mice treated with 2 × 5 mg/kg/d artesunate (p < 0.05) or artemisone (both doses; p < 0.001). The effects of 2 × 2.5 mg/kg/d artesunate or artemisone were similar, but artemisone was significantly more effective than artesunate, at the higher dose (p < 0.001). Average values ± SD (error bars) are presented. Arrows represent injections.

In the case of human Plasmodium infection, early detection of blood parasites (before symptoms are evident) is unlikely. Therefore the effect of delayed treatment was examined using artesunate, shown to be more effective than quinine or quinidine (both recommended as standard therapy for severe anaemic malaria in the United States) [61] and artemisone, the most recent artemisinin derivative approved for clinical trials [60]. The drugs were administered i.p. in 20 μl DMSO as a split daily dose on days 7-9 post-inoculation, to C57Bl/6 mice infected with PbAus. All control mice died of ECM by day 12 post-inoculation. Following a secondary rise in parasitaemia after treatment, the mice were again injected with artesunate or artemisone, on days 20-22 post-inoculation. Figure 5 demonstrates the results of artemisone vs. artesunate therapy. Administration of 2 × 2.5 mg/kg/d artesunate slightly delayed but in general did not prevent ECM death (p = 0.4); 2 × 5 mg/kg/d artesunate prevented ECM in most mice (p < 0.0001) and delayed death from anemic malaria by approximately one week (p < 0.0001). In contrast, both doses of artemisone prevented ECM in all treated mice, delayed recrudescence and prevented death (in 5/8 and 8/8 mice for 2 × 2.5 mg/kg/d and 2 × 5 mg/kg/d; p < 0.0001 for treated vs. control mice and for 2 × 2.5 vs. 2 × 5 mg/kg/d artemisone, respectively; Figure 5A, Table 4). 2 × 2.5 mg/kg/d artemisone was more effective in delaying mouse death than the higher dose of artesunate tested (p < 0.01). Changes in mouse temperature (Figure 5B) and weight (Figure 5C) were in accordance with disease severity, and the differences in overall group scores (p < 0.01). Figure 6 clearly demonstrate the superior effect of artemisone over artesunate.

**Table 4:** Disease pattern in PbAus-infected C57BL/6 mice treated with artesunate or artemisone twice a day, on days 7-9 post-inoculation.

Compound	Dose	Infection outcome		
		Cerebral malaria	Anemic malaria	Cured
DMSO	20 μl	4/8	4/8	0/8
Artesunate	2 × 2.5 mg/kg/d	6/8	2/8	0/8
	2 × 5 mg/kg/d	1/8*	7/8*	0/8
Artemisone	2 × 2.5 mg/kg/d	0/8*	3/8	5/8*^†^
	2 × 5 mg/kg/d	0/8*	0/8*^†^	8/8*^†^

*Significant vs. control.

^†^Significant vs. artesunate.

**Figure 7:**
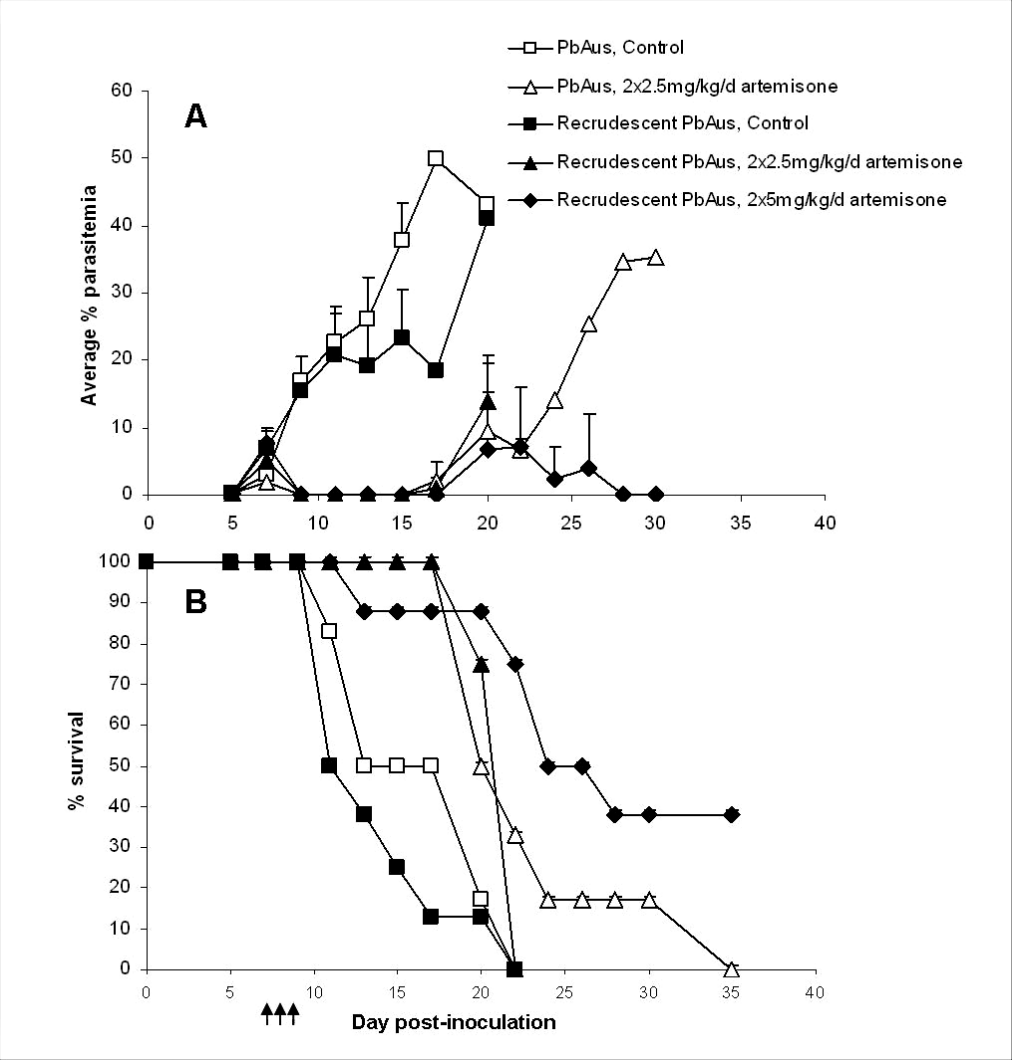
**Average parasitemias (A) and survival (B) of C57BL/6 mice infected with recrudescent PbAus, or PbAus, and administered artemisone twice a day, on days 7-9 post-inoculation**. Recrudescent parasites were isolated from peripheral blood of C57Bl/6 mice previously treated with 2 × 5 mg/kg/d artemisone, and were injected to naïve mice. After an additional passage, the recrudescent parasites were injected to C57Bl/6 mice, and artemisone administered at 2 × 2.5 mg/kg/d. Two additional groups of mice were infected with non-recrudescent PbAus and similarly treated. No significant differences were seen in either parasitemias or survival when comparing control PbAus- and recrudescent PbAus-infected mice. Differences in parasitemias between control and treated groups were significant (p < 0.001), as were the differences in survival rates. Average values ± SD (error bars) are presented. Arrows represent injections.

The major limitation of artemisinin-type drugs is their short half-life, which necessitates frequent administration; this leads to non-compliance, recrudescence, and possibly the development of resistance [24,62]. As a result, the World Health Organization recommends that these drugs be administered together with a long half-life anti-plasmodial drug (artemisinin combination therapy; ACT). Our experiments examined whether recrudescent parasites that appeared following artemisone therapy displayed any drug resistance. Recrudescent parasites (referred to as 'recrudescent PbAus') were isolated from peripheral blood of C57Bl/6 mice, which had previously undergone a three-day treatment with 2 × 5 mg/kg/d artemisone, and were injected to naïve mice. After an additional passage, the recrudescent PbAus parasites were injected to C57Bl/6 mice, and artemisone administered at 2 × 2.5 mg/kg/d; control mice were administered DMSO. Two additional groups of mice were infected with non-recrudescent PbAus and similarly injected with artemisone or DMSO. The results demonstrate a lack of parasite resistance to artemisone (Figure 7). No significant differences in parasitaemia were seen when comparing the two control groups. Administration of 2 × 2.5 mg/kg/d artemisone caused a similar, rapid drop in parasitaemia in both PbAus- and recrudescent PbAus-infected mice (Figure 7A). No significant difference in survival was seen when comparing the two control groups (p = 0.3), and when comparing treated groups (Figure 7B). An increased dose of 2 × 5 mg/kg/d led to complete cure in 3/7 mice (Figure 7B).

**Figure 8:**
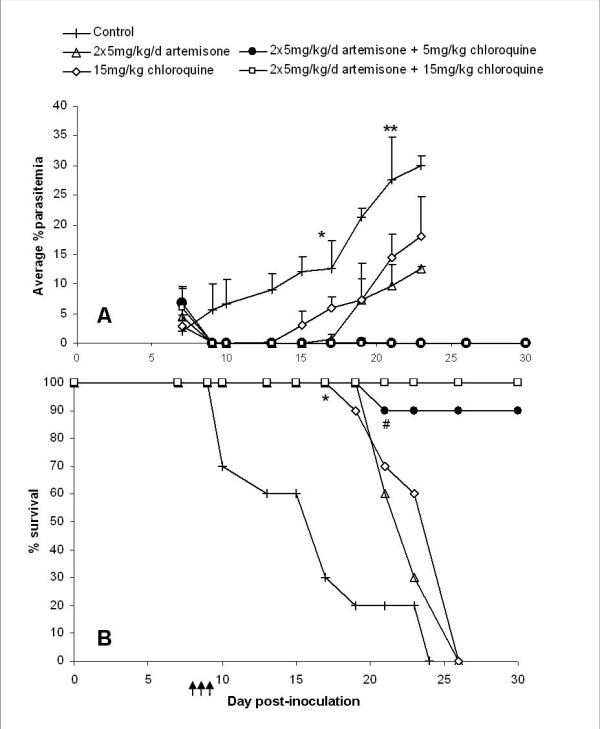
**Average parasitemias (A) and survival (B) of C57BL/6 mice infected with PbAus and administered artemisone or chloroquine monotherapy, or combination therapy**. The efficiency of artemisone-chloroquine combination therapy, to prevent the cerebral symptoms and achieve complete parasite clearance, was examined. Mice were treated on days 7-9 post-inoculation (n = 7 mice per group); control mice (n = 10) were administered saline. On day 19 post-inoculation, 2 control mice had not yet succumbed to the disease, vs. 6 mice administered chloroquine monotherapy, and 7 mice in each of the remaining treated groups. Although delayed mortality was seen in all treated groups (p < 0.05), chloroquine or artemisone monotherapy did not lead to complete cure. In contrast, all but one mouse treated by combination therapy were completely cured.*p < 0.001, all groups. **p < 0.05, control vs. monotherapy groups. ^#^p < 0.05, monotherapy vs. combination therapy groups. Arrows represent injections. Average values ± SD (error bars) are presented.

**Figure 9:**
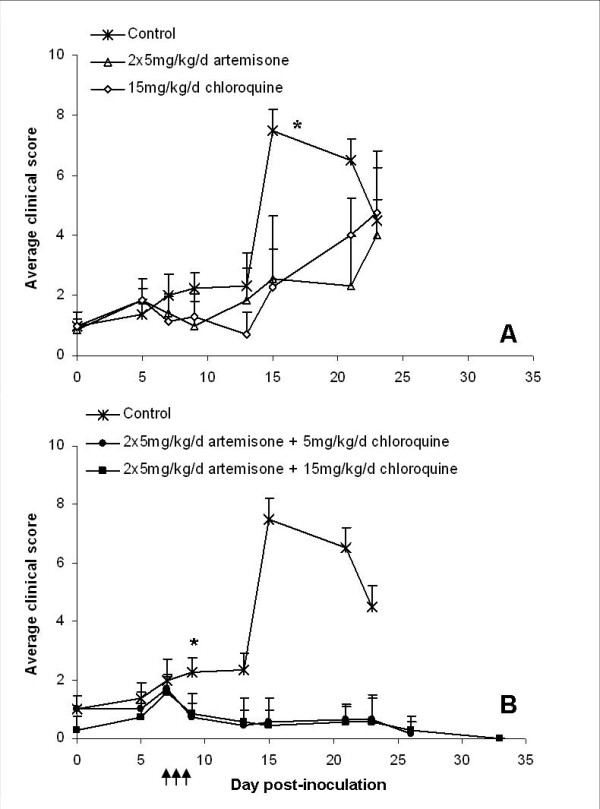
**Average clinical scores of C57BL/6 mice infected with PbAus and administered artemisone or chloroquine monotherapy (A), or combination therapy (B)**. No significant difference in clinical score was seen during the ECM phase when comparing control, artemisone- and chloroquine-treated groups. In contrast, significantly lower scores were seen in mice treated with artemisone and chloroquine throughout the experiment. *p < 0.05, control vs. treated groups. Average values ± SD (error bars) are presented. Arrows represent injections.

A further experiment was performed in order to examine the efficiency of artemisone-chloroquine combination therapy, to prevent the cerebral symptoms and achieve complete parasite clearance (Figure 8). C57Bl/6 mice were infected with PbAus as previously described, and treated on days 7-9 post-inoculation with 2 × 5 mg/kg/d artemisone, 15 mg/kg/d chloroquine, or a combination of 2 × 5 mg/kg/d artemisone and 5 or 15 mg/kg/d chloroquine. 80% of control mice died of ECM, whereas monotherapy with either drug caused a drop to non-detectable parasitaemia levels by day 10 post-inoculation (Figure 8A). Administration of artemisone was more effective than chloroquine treatment: parasitaemia levels in all artemisone-treated mice were below detection by the end of treatment, and recrudescence was observed starting on day 15 post-inoculation. In contrast, parasitaemia levels of mice administered chloroquine dropped to zero only on day 10, and recrudescence was seen starting on day 13 post-inoculation. Although delayed mortality was seen in all treated groups (p < 0.05) (Figure 8B), chloroquine or artemisone monotherapy did not lead to complete cure. The majority of mice, in both cases, succumbed to ECM, and the rest died later at high parasitaemia. Except for one, all mice treated by combination therapy were completely cured. No significant difference in clinical score was seen during the ECM phase when comparing control, artemisone- and chloroquine-treated groups (Figure 9A). In contrast, significantly lower scores were seen in mice treated with artemisone and chloroquine throughout the experiment (Figure 9B).

## Discussion

In this research, the efficacy of several artemisinin derivatives was examined for treatment of ECM in ICR and C57BL mice. The most widely used model for CM utilizes inbred (C57BL) mice. The decision to use genetically heterogenous ICR mice in our research, as an additional model for CM, was made in order to enable a more accurate reflection of human CM susceptibility and the possible effect of treatment. The validity of ECM in outbred mice as a model for CM has previously been demonstrated [41,63-65]. Artemiside and artemisone were shown to be more effective than DHA and artesunate, two artemisinin derivatives used in anti-malarial artemisinin-based combination therapy. In all experiments, treatment with artemisone led to complete cure, or reduced parasitaemia to undetectable levels; recrudescence was followed by death from either anaemic or cerebral malaria. In these cases, the significant delay to the time of death from anaemic malaria, or to the appearance of cerebral symptoms, may open a considerable time window for adequate treatment. A steady state latency, in which non-detectable levels of parasites remain in the blood, has been described in cases of human *P. falciparum *infection [65]. As demonstrated, the recrudescent parasites could also be completely eliminated by repeating the artemisone treatment.

The results with artemisone demonstrate that complete cure may be achieved even when treatment is started at late stages of pathogenesis, 6 days post-inoculation. These findings are especially important, as human malaria is diagnosed only after clinical symptoms are apparent, and especially so if the rapid process of CM has been initiated. Furthermore, co-administration of artemisone with low doses of chloroquine prevented recrudescence and led to complete cure in all mice. Recrudescence that appeared after monotherapy did not cause any short-term drug resistance, as parasites isolated from the recrudescent mice were fully susceptible to artemisone.

The successful use of artemisone in combination with chloroquine, to which most strains of *P. falciparum *are resistant, may be analogous to the combination of proguanil and atovaquone (Malarone^®^). Although *P. falciparum *is resistant to both atovaquone and proguanil, the parasite is sensitive to Malarone^®^, which is used for both prophylaxis and treatment. Similarly, combination of artemisone with a partner drug to which resistance has developed may enable the latters reintroduction.

Although slight antagonism between artemisone and chloroquine has been reported in in vitro tests against *P. falciparum *[66], synergism or no interaction has been observed when the drugs were tested in vivo against *Plasmodium yoelii *NS or *P. berghei *NY, respectively. The in vivo experiments were based on the Peter's four-day test, in which mice are treated on the first day post-inoculation and for an additional three days thereafter; the analysis of results is based on blood smears taken on day 5 post-inoculation. This approach does not reflect the final results of the treatment (i.e. the fate of the animals) or provide any indication of the immune responses of the infected animals. Therefore, the Peter's four-day test is inadequate for estimation of the effect of drug treatment on severe (cerebral) malaria, where pathogenesis is most pronounced a week or more after infection and pathology (or lack of pathology) is the result of a prolonged innate immune response [34] and early acquired immunity.

Artemiside appeared to be even more effective than artemisone, but this substance has yet to be submitted to preclinical toxicological evaluation. Nevertheless, although it does display neurotoxicity in in vitro assays with neuronal cell lines [34], this is substantially less than that of DHA. It is approximately three times more active than artemisone in vitro against *P. falciparum*, and its therapeutic index suggests that it will be usable in a clinical setting. In principle it may act as a prodrug for artemisone through oxidation of the thiomorpholine ring to the S, S-dioxide of artemisone.

## Conclusions

The results show that artemiside and artemisone are the two most effective artemisinin derivatives. Administration of artemisone may result in complete cure even when treatment is started at late stages of pathogenesis; recrudescence that appeared after monotherapy did not cause any short-term drug resistance. Co-administration of artemisone with low doses of chloroquine prevented recrudescence and led to complete cure in all mice. Combination of artemisone with a partner drug to which resistance has developed may enable reintroduction of the latter. Artemiside, found even more effective than artemisone, may represent a new option for artemisinin-based anti-malarial therapy.

## Competing interests

The authors declare that they have no competing interests.

## Authors' contributions

JHWG participated in experiments and preparation of the manuscript. ABM, JAMQ, HWC and WCC participated in experiments. NH and RKH supervised the study and participated in preparation of the manuscript. JG and YB initiated and coordinated the study. All authors read and approved the final manuscript.
